# Usefulness of Eurasian Magpies (*Pica pica*) for West Nile virus Surveillance in Non-Endemic and Endemic Situations

**DOI:** 10.3390/v11080716

**Published:** 2019-08-05

**Authors:** Sebastian Napp, Tomás Montalvo, César Piñol-Baena, Maria Belén Gómez-Martín, Olga Nicolás-Francisco, Mercè Soler, Núria Busquets

**Affiliations:** 1 IRTA, Centre de Recerca en Sanitat Animal (CReSA IRTA-UAB), 08193 Bellaterra, Spain; 2 Servei de Vigilància i Control de Plagues Urbanes, Agencia de Salud Pública de Barcelona, 08023 Barcelona, Spain; 3 CIBER Epidemiologia y Salud Pública, CIBERESP, Instituto de Salud Carlos III, 28029 Madrid, Spain; 4 Departament de Territori i Sostenibilitat, Centre de Fauna de Vallcalent, 25199 Lleida, Spain; 5 Laboratorio Central de Veterinaria, Ministerio de Agricultura, Pesca y Alimentación (MAPA), 28110 Algete (Madrid), Spain; 6 Departament d’Agricultura, Ramaderia, Pesca i Alimentació Generalitat de Catalunya, Servei de Prevenció en Salut Animal, 08007 Barcelona, Spain

**Keywords:** West Nile virus, Eurasian magpies, wild birds, sentinels, surveillance

## Abstract

In September 2017, passive surveillance allowed the detection of West Nile virus (WNV) lineage 2 for the first time in northern Spain in a northern goshawk (*Accipiter gentilis*). However, a cross sectional study carried out in Eurasian magpies (*Pica pica*) in a nearby area evidenced that WNV had been circulating two months earlier. Therefore, active surveillance in Eurasian magpies proved its effectiveness for the early detection of WNV in a non-endemic area. Further surveys in 2018 and the beginning of 2019 using young magpies (i.e., born after 2017) showed the repeated circulation of WNV in the same region in the following transmission season. Therefore, active surveillance in Eurasian magpies as well proved to be useful for the detection of WNV circulation in areas that may be considered as endemic. In this manuscript we present the results of those studies and discuss reasons that make the Eurasian magpies an ideal species for the surveillance of WNV, both in endemic and non-endemic areas.

## 1. Introduction

West Nile virus (WNV) belongs to the genus *Flavivirus* within the family *Flaviviridae*. The natural infection cycle involves mosquitoes, primarily *Culex* spp. and birds, while horses and humans are considered dead-end hosts. It is regarded as the most widespread arbovirus in the world and has circulated in Europe for decades, where it has significantly expanded in recent years. In fact, 2018 was the year with the highest number of autochthonous infections reported in humans in the European Union and neighboring countries (2083), a 7.2 fold increase in relation to 2017, while the number of outbreaks in horses increased by 30% [1].

Timely detection of WNV circulation is essential for the effective implementation of measures such as safety procedures on blood collected for donations, vector control and communication to the relevant Animal and Public Health authorities as well as to the public [2]. In Europe, while the lineage 2 WNV strain was able to cause significant mortality in several wild bird species, mainly in goshawks (*Accipiter gentilis*) [3], the lineage 1 (Mediterranean/Kenyan cluster) was characterized by limited or no pathogenicity for birds [4]. Therefore, it is recommended to combine the passive surveillance in wild birds with some sort of active surveillance [5]. The use of sentinel birds has been proposed as an effective strategy for the early detection of WNV circulation and for the identification of affected areas [2,6]. Since a huge number of bird species from several different orders are considered susceptible to WNV infection [7], selection of the ideal sentinel bird species for a given area is difficult [8]. Passerine birds, and in particular the Corvidae family, are particularly susceptible to WNV infection [9,10]. Eurasian magpies (*Pica pica*) have been effectively used for the detection of WNV circulation in endemic areas, and the fact that this species is sedentary allows the evaluation of WNV circulation in a particular region. In France, after the 2004 epidemics in horses in the Camargue, a cloacal sample from a seropositive juvenile magpie sampled in 2005 tested positive by nested RT-PCR, indicating that WNV circulated among wild birds in that year [11]. In Greece, the analysis of tissues (kidney, heart and liver) from Eurasian magpies hunted at the time of the 2010 human WNV epidemic in central Macedonia allowed the molecular identification and characterization of a WNV lineage 2 strain [12]. 

In 2017, despite more than 10 years of intensive surveillance, local WNV circulation in Catalonia (north-eastern Spain) had not been demonstrated. However, WNV positivity by both competition ELISA (cELISA) and the serum neutralization test (SNT) had been consistently detected, mostly in a particular area in western-central Catalonia (Segrià region), but always in wild bird species that could have migrated from other areas [13]. With the aim of evaluating whether local WNV transmission occurred in that area, a series of cross-sectional surveys in Eurasian magpies were carried out in 2017. After WNV lineage 2 was isolated in 2017 from a northern goshawk in a nearby location, cross-sectional surveys in Eurasian magpies were repeated in 2018 and in the beginning of 2019, to determine whether the virus circulated also in 2018. We evaluate the results of those cross-sectional studies, and discuss the advantages of using Eurasian magpies for WNV surveillance in non-endemic and endemic situations.

## 2. Materials and Methods

### Study Design

A multiyear series of cross-sectional surveys for WNV were carried out in Eurasian magpies. Magpies were actively captured from the area of Torre Ribera, located 5 Km west of the city of Lleida, by personnel of the Department of Territory and Sustainability as part of the measures implemented to protect the Lesser Grey Shrike (*Lanius minor*). This species is the scarcest vertebrate in the Iberian Peninsula, and it is currently classified as “critically endangered” under the International Union for Conservation of Nature (IUCN) criteria [14]. Because of that, the Department of Territory and Sustainability implements a conservation plan in Torre Ribera, where its main population is located (13 animals in the last count in 2017) [14]. The plan includes providing additional feeding opportunities (insects), management of nesting habitats and control of predators (Eurasian magpies).

Testing of WNV in Eurasian magpies in this area was considered interesting because WNV circulation was suspected but could never be proven, as several positives for WNV both by cELISA and SNT had been detected over the years, but never in resident birds [13].

Captures were carried out using a wire net trap, which contained a Eurasian magpie as a lure. Blood samples were collected from the brachial vein or the heart. The birds were euthanized in a tight container filled with CO_2_ or with Dolethal (sodium pentobarbital) administered intravenously. Serum samples were tested by a competition ELISA (cELISA, IDvet-ID Screen^®^ West Nile Competition, Montpellier, France) and positive samples were sent to the Central Veterinary Laboratory (CVL) in Algete for confirmation by SNT for WNV and Bagaza virus (BAGV) [15], both of which have been detected in birds in Spain. In the SNT, samples were analyzed in duplicate and the 50% neutralizing dose (ND50) for each serum was calculated according to the Spearman Kärber Method [16]. This method allows determining the antibody concentration that inhibited the 90% virus-induced cytopathic effect in at least 50% of the wells. 

**Figure 1:**
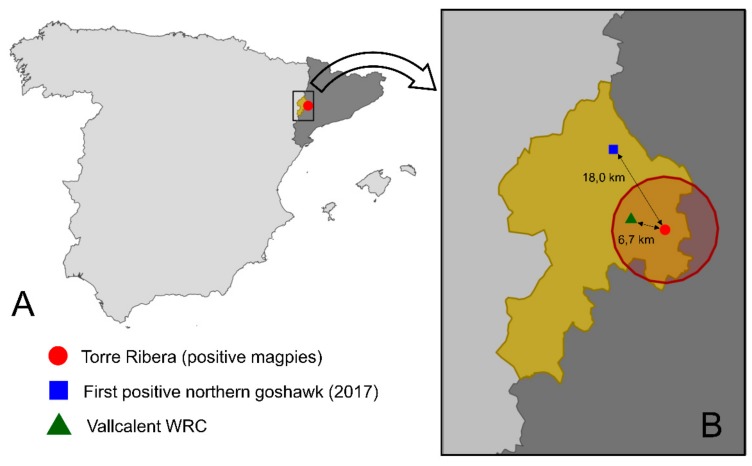
(**A**) Location of Torre Ribera, the magpies sampling area (red point) within Segrià region (in gold), Catalonia (dark grey) and Spain (light grey). (**B**) Zoomed area with Torre Ribera sampling area (red point), 10 km buffer area (dark red circle), location of the first positive northern goshawk (blue square) and Vallcalent Wildlife Recovery Center (WRC) (green triangle).

In 2017, between the 26th of April and the 4th of August, a total of 89 magpies were captured on 24 different days. Cross-sectional surveys were repeated in 2018, after the detection of WNV in Catalonia for the first time in September 2017 [17] in two different locations just a few kilometers from Torre Ribera (Figure 1). Between the 23rd of April and the 28th of July 2018, 83 magpies were captured on 34 different days. In 2019, between the 6th of March and the 30th of April (i.e., before the start of the WNV transmission season), 35 magpies were captured on 12 different days.

Since June 2018 the information on the age of the animals started to be recorded. Magpies were classified, according to Svensson [18], as age-category 3 (born in that year), age-category 5 (born in the previous year) and adults (born before the previous year), based on plumage characteristics. That allowed determining whether WNV circulated also in 2018 (as detection of WNV infection in age-category 3 in 2018, or age-category 5 early in 2019 would be indicative of WNV circulation in 2018). Furthermore, in 2018, eight magpies were caught in the Wildlife Recovery Center (WRC) of Vallcalent, in the city of Lleida, by personnel of Public Health Agency of Barcelona (ASPB) within the WNV Surveillance Program in Catalonia. 

In order to characterize the area of study, the types of physical coverage of the Earth’s surface in the circular area within a 10 km radius around Torre Ribera, were obtained from CORINE Land Cover [19]. Data extraction was carried out using R [20].

## 3. Results

Prior to the 28th of July 2017, all magpies (*n* = 74) sampled in Torre Ribera were negative for WNV by cELISA (Table 1). However, two of the magpies tested on the 28th of July were positive by cELISA, one of which was later confirmed by SNT with a 10 titer. Two further samples collected on the 4th of August were positive by cELISA, and were confirmed by SNT in Algete with titers of 10 and 20.

In 2018, after the detection of WNV the year before, eighty-three magpies from Torre Ribera and eight from Vallcalent WRC were sampled and tested for WNV antibodies (Table 2). Since the first surveys in April, results consistently showed high seroprevalences of flavivirus infections (i.e., positivity to cELISA) that ranged between 58% and 83%. Infection by WNV (i.e., positivity to cELISA and positivity for WNV by SNT) was also regularly detected, although monthly prevalences were much lower, between 12% and 17%. SNT titers ranged between 10 and 60. Interestingly, one of the magpies sampled in July 2018, which was found positive by cELISA and positive for WNV by SNT (with a titer of 20), was an age-category three (i.e., born in the spring of 2018 and therefore only a few months old). Besides, one of the magpies sampled in June 2018 that was positive by cELISA, tested positive for Bagaza virus (BAGV) by SNT (with a titer of 30).

In 2019, fourteen of the thirty-five (i.e., 40%) magpies sampled in March and April in Torre Ribera were positive by cELISA. Six of those fourteen magpies were also positive for WNV by SNT, and five of them were age-category five (i.e., born in 2018).

We defined an area of study of 10-km area around Torre Ribera, which also included Vallcalent WRC (Figure 1), where circulation of flaviviruses and specifically of WNV, had been demonstrated. In that area, we evaluated the ecological features and the results indicated that ninety percent was comprised of agricultural areas, of which half were permanently irrigated arable land and 30% were areas of fruit trees. Another 7% percent of the study area contained artificial surfaces, mainly urban areas (dominated by dwellings and buildings), but also industrial and commercial zones.

## 4. Discussion

In September 2017, samples of nervous tissue of a northern goshawk that had been found sick a few kilometers north of the city of Lleida and was transferred to Vallcalent WRC (Figure 1), tested positive for WNV infection by RT-qPCR [17]. The strain was identified as belonging to lineage 2, and the phylogenetic analyses showed that it was related to the Central/Southern European strains. Shortly after the first case, positivity for WNV (by cELISA and SNT) was detected in other wild bird species held in Vallcalent WRC including two other northern goshawks and 13 bearded vultures (*Gypaetus barbatus*). To evaluate the extent of WNV circulation, in October 2017, a cross-sectional survey was carried out in poultry and horses in the area (10 km radius) around the first case. Widespread circulation of WNV was evidenced within this area as five out of the eight chicken holdings and three out of the seven horses holdings tested positive for WNV (by both cELISA and SNT) [17]. This was the first time WNV circulation was reported in the north of the Iberian Peninsula and it evidenced the westward spread of WNV lineage 2, a strain that had been restricted to central and south-eastern Europe where it caused hundreds of human cases [21]. Until then, only WNV lineage 1 had been reported in Spain [22].

The fact that all 74 magpies tested in Torre Ribera before the 28th of July 2017 were negative, while positives for WNV (by both cELISA and SNT) were consistently detected afterwards, seems to indicate that the introduction of the virus into the area occurred around that date. Interestingly, the first confirmed case of WNV in Catalonia (the PCR-positive northern goshawk) was found on the 24th of September at only 18 km from Torre Ribera. That means that active surveillance in magpies allowed the detection of WNV circulation almost two months earlier than any other component of the surveillance system, evidencing its usefulness for early WNV detection in non-endemic areas. Active surveillance in wild birds is expensive and logistically demanding and therefore, the selection of the target area is critical. We chose an area where we had positivity for WNV by cELISA and SNT in migratory birds throughout the years but where local circulation could not be demonstrated [13]. Moreover, the area has one of the highest densities of Eurasian magpies in Catalonia [23]. Because magpies predate on eggs and chicks of songbirds and gamebirds, they are considered as a harmful bird species, and are frequently subject to population control in Europe [24] which may be used for WNV surveillance. In our case, we took advantage of the program for the protection of the Lesser Grey Shrike for obtaining the samples from magpies.

In 2018, even from the first surveys, the results indicated intense circulation of flaviviruses and specifically of WNV in the area. As the earliest surveys in 2018 took place at the beginning of the mosquito transmission season, positivity is likely to have been the result of the transmission in 2017. From 2018 onwards, 64% of the Eurasian magpies tested in the study area were positive for flaviviruses (i.e., cELISA positive) and 15% were confirmed as positives for WNV by SNT. These results suggest that the cELISA used allowed the detection of flaviviruses different to WNV that were circulating in the studied area, which is in agreement with a recent study that has demonstrated the presence of cross-reactions between WNV and other flaviviruses such as BAGV or Usutu virus (USUV) when the IDvet kit was used [25]. In fact, antibodies against BAGV were detected by SNT in a magpie sampled in June 2018, which is the first time in this bird species. BAGV emerged in southwestern Spain in 2010 causing high mortality rates, particularly in red-legged partridges [26]. In 2017, antibodies against BAGV were detected in healthy poultry in Catalonia [17] indicating a more extensive distribution of BAGV in Spain. The detection of BAGV antibodies in the present study supports this wider circulation of BAGV. Even though it is known that BAGV is able to cause severe disease in some bird species such as grey partridges [27], the susceptibility of other wild birds, including Eurasian magpies remains scarcely studied. In order to understand the epidemiology of BAGV, more studies on the potential reservoirs of this virus are needed. Similarly, circulation of USUV, since its first emergence in Europe, in Italy in 1996 [28], has been demonstrated in several central and western European countries, causing in some cases, significant mortality in several wild bird species such as common blackbirds (*Turdus merula*) [29]. Actually, there are previous reports of USUV circulation in Catalonia, as in August 2006 the virus was isolated from a *Cx. pipiens* mosquito [30]. The results obtained in magpies are in agreement with those of the cross-sectional survey carried out in poultry and horses in 2017 after WNV confirmation, which not only evidenced circulation of WNV but also other flaviviruses in the area surrounding Torre Ribera [17]. The identity of the flaviviruses circulating in the region, and their effect on WNV transmission deserves further attention.

The reasons why WNV transmission occurred in the study area are not known yet, but the ecological assessment indicated that the study region consists of agricultural areas, mainly permanently irrigated arable land, which has been associated with an increased risk of WNV transmission because of its favorable conditions for the development of the larval stages of *Culex* spp. mosquitoes [31].

The possibility of determining the age of magpies allows their use to evaluate whether recent transmission of WNV has occurred in an area where the virus is endemic. Since the 28th of May 2018, the age of magpies sampled started to be recorded, but as the vast majority of them were adult animals (i.e., born before 2018) it was impossible to differentiate whether the infections were the result of transmission in 2018 or in the previous years. However, the fact that positivity for WNV by SNT was detected in an age-category 3 magpie sampled on July 2018 suggests WNV circulation in the area also in 2018. That finding was further supported by the detection of other five age-category 5 magpies positive for WNV by SNT among those sampled in March and April 2019 (i.e., before the start of the 2019 WNV mosquito transmission season). Therefore, active surveillance in young magpies also allows detection of recent WNV circulation in endemic areas. Although a new introduction of WNV in 2018 cannot be ruled out, the fact that infection occurred in exactly the same area as 2017 leads us to think that WNV overwintering may have occurred. WNV persistence throughout the winter season in Europe has been demonstrated by WNV lineage 2 RNA detection in overwintering mosquitoes [32]. WNV persistence in tissues of infected birds combined with predation or scavenging behavior offers an alternative hypothesis for overwintering. Alternatively, WNV positivity in juvenile birds may be explained by the passive transfer of maternal antibodies as it has been demonstrated in different bird species [33,34,35]. However, maternal antibodies in those studies seemed to be short-lived, in particular in the passerine house sparrows (*Passer domesticus*), in which no antibodies were detected after nine days post-hatch. Besides the detection in Eurasian magpies, no WNV circulation was reported in the Segrià area in 2018. In fact, the only three WNV outbreaks reported in Catalonia in 2018 were in horses and were more than a hundred kilometers to the east [36]. Unfortunately, we could not isolate the virus in 2018 from either birds or horses, and the serological tests do not allow differentiating between lineage 1 and lineage 2.

Other advantage of Eurasian magpies as sentinels is that they have a very broad distribution covering most of Europe and Asia [37]. Moreover, they are one of the most abundant corvid species in Europe, with an estimated stable population of between 10.3 and 17.8 million pairs [37]. In Catalonia, the population is estimated to be between 206,000 and 286,000 adult individuals [23]. Furthermore, both in Spain and in central Europe adult magpies are considered sedentary, while the dispersion of juveniles is limited and does not extend beyond 30–40 km from the place of birth [38,39]. Sedentary behavior of magpies makes them ideal to evaluate local WNV transmission in a particular area. Furthermore, their gregarious behavior and the existence of effective capture methods make them relatively easy to catch. Additionally, besides agricultural areas, magpies also breed within urban areas [40,41,42] allowing the assessment of the risk of WNV infection in human-inhabited areas where not many bird species are available for surveillance. 

A key argument in favor of using magpies as sentinels is that they seem to have a higher prevalence of WNV antibodies than other sedentary bird species, although it is not clear why they are so frequently exposed [11]. The fact that *Cx. pipiens*, the main vector of WNV in Europe, seems to have a significant feeding preference for magpies (i.e., *Cx. pipiens* are fed upon magpies more frequently than it would correspond for its density) is likely to contribute to their infection, in particular, in peridomestic areas [43]. In addition, magpies are altricial (i.e., hatched in an undeveloped state), and the low feather coverage in altricial species has been associated with an increased risk of WNV infection [44]. On the other hand, experimental infections of Eurasian magpies with WNV lineage 1 and 2 strains, evidenced the presence of infectious virus and viral RNA in feather pulps as well as in oral and cloacal swabs [45]. That may represent a source of WNV horizontal oral transmission which would be facilitated by the gregariousness of magpies. Using a deterministic transmission model, Montecino—Latorre and Barker [46] found that winter crow roosts could allow for WNV persistence through the winter, with fecal-oral transmission as the main crow-to-crow transmission pathway.

In addition, WNV can be detected in several tissues of a wide range of bird species experimentally infected with the virus [9], which may serve as a source of virus for scavenging species such as Eurasian magpies. A recent study of the feeding habits of magpies in agricultural environments of central Spain showed that a relatively high proportion of consumptions during the breeding season (12.7%) corresponded to passerines [24] that are highly competent for WNV (see Komar et al. [9]). It was suggested that magpie predation on birds might be opportunistic and occur mainly during the breeding season (i.e., spring) as a result of the increase of the predatory pressure on birds when invertebrates, the main animal component of their diet, are less available. 

Besides their susceptibility to WNV infection, which makes them ideal as sentinels, magpies may be considered as highly competent for WNV transmission, another reason for using this species for WNV surveillance. Experimental infections of Eurasian magpies with lineages 1 and 2 currently circulating in Europe, demonstrated that in the majority of animals the level of viraemia reached was well above what is considered to be needed to infect the main vector species [45]. That is consistent with experimental infections carried out in other species of the Corvidae family such as Blue Jay (*Cyanocitta cristata*), American Crow (*Corvus brachyrhynchos*) and Black-Billed Magpie (*Pica hudsonia*), which proved they are also highly competent [9]. Considering the competence of magpies, their preference for peridomestic areas and the feeding preference of *Cx. pipiens* for magpies, they may play an important role as bridge-host for WNV transmission to humans.

For all of the above reasons, Eurasian magpies are an ideal species to be used as sentinels for the surveillance of WNV. They may allow the early detection of WNV, both in non-endemic, as well as in endemic areas. However, when considering WNV surveillance in endemic areas, and in particular if the objective is early detection of viral circulation, to implement control measures, sampling should target age-category three magpies, that will allow us to discriminate between recent infection (i.e., within that season) and infection on the previous years.

Besides Eurasian magpies, there are other wild bird species that may be effectively used for WNV surveillance such as the house sparrow (*Passer domesticus*) [47]. House sparrows are abundant, have a worldwide distribution, they share habitats with humans, and they are known to be susceptible to WNV infection [48,49]. However, in comparison with Eurasian magpies, house sparrows are not as attractive for *Cx. pipiens* [43] and they lack the scavenging behavior of magpies that may play a relevant role for WNV transmission. Domestic chickens have also proven their efficiency as sentinels for WNV in several countries [2] although the implementation of this type of surveillance involves some logistic effort for the maintenance of the animals.

## Figures and Tables

**Table 1:** Results of the cross-sectional serosurveys carried out in magpies in 2017 for the detection of West Nile virus (WNV) infection.

Month (2017)	Tested	cELISA Positive	Seroprevalence Flavivirus (%)	WNV SNT Positive	WNV SNT Titers	Seroprevalence WNV (%)
**April**	10	0	0	0	-	0
**May**	39	0	0	0	-	0
**June**	13	0	0	0	-	0
**July**	18	2	11	1	10	6
**August**	9	2	22	2	10–20	22

**Table 2:** Results of the cross-sectional serosurveys carried out in magpies in 2018 and early 2019 for the detection of WNV infection.

Month	Tested	cELISA Positive	Seroprevalence Flavivirus (%)	WNV SNT Positive	WNV SNT Titers	Seroprevalence WNV (%)
**April 2018**	24	20	83	4	10–60	17
**May 2018**	29	24	83	4	10	14
**June 2018**	26	15	58	3 *	10–40	12
**July 2018**	12	8	67	2	20 ^‡^	17
**March 2019**	26	10	38	2	40 ^†^	8
**April 2019**	9	4	44	4	10–40 ^††^	44

* Besides the three animals positive for WNV by SNT, there was one animal positive for Bagaza virus (BAGV) by SNT (titer 30). ^‡^ One age-category three magpie (i.e., born in 2018). ^†^ One age-category five magpie (i.e., born in 2018). ^††^ All four age-category five magpie (i.e., born in 2018).
